# ClusterVAP: study protocol for multicentre proteomic endotyping of ventilator-associated pneumonia

**DOI:** 10.1136/bmjresp-2025-003830

**Published:** 2026-03-09

**Authors:** Fredrik Sjövall, Pedro Póvoa, Johan Petersson, Despoina Koulenti, Ana Catalina Hernandez Padilla, Julien Vaidie, Alicia Lind, Frederik Boëtius Hertz, Magnus Paulsson

**Affiliations:** 1 Department of Intensive Care and Perioperative Medicine, Skåne University Hospital, Malmö, Sweden; 2 Hospital de São Francisco Xavier, Lisboa, Portugal; 3 NOVA Medical School, NOVA, Lisbon, Portugal; 4 Function Perioperative Medicine and Intensive Care, Karolinska University Hospital, Stockholm, Sweden; 5 Section of Anaesthesiology & Intensive Care Medicine, Department of Physiology and Pharmacology, Karolinska Institutet, Stockholm, Sweden; 6 Critical Care Department, King’s College Hospital NHS Foundation Trust, London, UK; 7 UQ Centre for Clinical Research, The University of Queensland, Brisbane, Queensland, Australia; 8 Inserm CIC 1435 and Réanimation polyvalente, CHU Limoges, Limoges, France; 9 UMR INSERM 1092, Universite de Limoges, Limoges, France; 10 Service de Réanimation Polyvalente, CHU Limoges, Limoges, France; 11 Department of Diagnostics and Intervention and Umeå Centre for Microbial Research, Umeå University, Umeå, Sweden; 12 Department of Clinical Microbiology, Copenhagen University Hospital, Rigshospitalet, Copenhagen, Denmark; 13 Department of Immunology and Microbiology, University of Copenhagen, Copenhagen, Denmark; 14 Infection Medicine, IKVL, Medical faculty, Lund University, Lund, Sweden; 15 Clinical Microbiology, Skåne University Hospital, Lund, Sweden

**Keywords:** Pneumonia, Critical Care, Respiratory Infection

## Abstract

**Introduction:**

Ventilator-associated pneumonia (VAP) is the most frequent healthcare-associated infection in intensive care units and is associated with high morbidity and mortality. Current diagnostic criteria lack specificity, leading to misclassification and unnecessary antibiotic use. Identifying patient subgroups with a common pathophysiological basis (pneumoclusters) may distinguish true VAP of varying aetiology and severity from non-infectious mimics, enabling more targeted therapy and improved antimicrobial stewardship.

**Methods and analysis:**

ClusterVAP is an exploratory, observational, prospective, multicentre cross-sectional study conducted in intensive care units across Sweden, France, Portugal, Denmark and the UK. Mechanically ventilated patients aged 18 years or older with newly developed clinical signs of lower respiratory tract infection will undergo bronchoalveolar lavage (BAL) or mini BAL sampling on clinical indication. Proteomic profiling using liquid chromatography tandem mass spectrometry will be performed on BAL supernatants. Unsupervised consensus clustering will define pneumoclusters, which will be characterised using clinical, microbiological and radiological data. 30-day outcomes, including mortality, ventilator-free days, antibiotic-free days, intensive care unit-free days and hospital-free days, will be compared across clusters to describe clinical trajectories. Candidate protein biomarkers for pragmatic cluster assignment will be derived using differential expression analysis.

**Ethics and dissemination:**

Ethical approval will be obtained at all participating sites. Deferred consent will be used where permitted, with subsequent patient or proxy consent according to local regulations. Results will be disseminated through peer-reviewed publications and scientific conferences.

**Trial registration number:**

NCT07245888.

**WHAT IS ALREADY KNOWN ON THIS TOPIC**

**WHAT THIS STUDY ADDS**

- ClusterVAP will apply proteomic analysis of bronchoalveolar lavage samples to identify distinct patient subgroups, termed pneumoclusters and derive candidate biomarkers for pragmatic classification. This approach aims to improve diagnostic precision and provide insight into the underlying pathophysiology of VAP.

**HOW THIS STUDY MIGHT AFFECT RESEARCH, PRACTICE OR POLICY**

- The findings may inform future diagnostic algorithms, support antimicrobial stewardship and guide personalised treatment strategies in critically ill patients with suspected VAP.

## Introduction

 Ventilator-associated pneumonia (VAP) is the most frequent healthcare-associated infection in intensive care units (ICUs) and remains a major contributor to morbidity and mortality among critically ill patients.^1 2^ Reported incidence estimates vary widely, reflecting differences in case mix and diagnostic definitions, but VAP is consistently identified as a leading infectious complication of invasive mechanical ventilation and a key determinant of clinical outcomes and resource use in intensive care.^1 2^

Despite its clinical importance, diagnosing VAP with confidence remains challenging. Routine practice relies on a combination of non-specific clinical features, new or worsening radiographic infiltrates, blood leucocyte counts and culture results from lower airway samples, none of which in isolation provides adequate diagnostic certainty.^3 4^ In a systematic review, the presence of an infiltrate on chest radiography demonstrated high sensitivity but very low specificity, and combining radiographic findings with one or more clinical or laboratory criteria did not substantially improve specificity in most combinations.^5^ As a result, a considerable proportion of patients who meet consensus clinical criteria for VAP do not have bacterial pneumonia when assessed against lung biopsy histopathology.^5^ Estimates suggest that up to one half of clinically diagnosed cases may represent alternative conditions that mimic infection, including ventilator-induced lung injury, cardiogenic pulmonary oedema or acute respiratory distress due to non-infectious causes, each of which requires different management.^3^ A consequence of misclassification is unnecessary antibiotic exposure or missed opportunities to deliver appropriate treatment for the condition.

Biomarker research has not yet closed this evidence gap. Multiple candidate markers measured in lower respiratory tract specimens have been evaluated, but none has achieved a level of accuracy that would support widespread clinical use. In the most comprehensive syntheses to date, individual biomarkers such as soluble triggering receptor expressed on myeloid cells show only moderate sensitivity and specificity, and overall the evidence base does not support adoption of any single marker for routine VAP diagnosis.^6 7^ The lack of a reliable biomarker panel for bedside application reinforces the need for alternative strategies that can improve the separation of true infection from non-infectious mimics and that can also account for the biological heterogeneity within VAP itself.

A further challenge lies in the marked heterogeneity of the VAP construct. Patients who satisfy broad clinical definitions likely comprise biologically distinct subgroups that differ in aetiology, host response, disease stage and prognosis. Grouping such patients under a single label obscures meaningful differences that could guide treatment selection, antimicrobial stewardship and trial design. Molecular endotyping offers a principled route to disentangle this heterogeneity by identifying patient subgroups with shared pathophysiology and clinical trajectory. Bronchoalveolar lavage (BAL) provides a sample from the lower respiratory tract that captures local host responses within the infected compartment, and proteomic profiling can quantify a wide range of host proteins that reflect ongoing inflammatory and immune processes. Integrating this molecular information with carefully curated clinical, microbiological and radiological data creates an opportunity to define coherent and clinically useful subgroups, here termed pneumoclusters, within the broad VAP syndrome.

The ClusterVAP study is designed to address these needs. It is an exploratory, observational, prospective, multicentre cross-sectional investigation conducted in ICUs in several European countries. Proteome-driven, unsupervised consensus clustering will be used to endotype patients into ‘pneumoclusters’ with distinct pathophysiology and clinical trajectories. By focusing on endotyping at the site of disease using proteomics, ClusterVAP aims to advance the field in three ways. First, it seeks to improve diagnostic discrimination between true infection and non-infectious mimics within the population currently labelled as suspected VAP, thereby reducing unnecessary antibiotic exposure and facilitating appropriate alternative management. Second, it aims to identify more homogeneous subgroups with shared pathophysiology and prognosis, which could enable personalised treatment strategies and provide a framework for targeted interventional trials. Third, it intends to generate parsimonious biomarker panels that, after validation, could be translated into practical tools for bedside classification. The present protocol sets out the rationale and objectives of the ClusterVAP study and outlines how proteome-driven clustering integrated with routinely collected data may fill gaps left by existing diagnostic methods.

## Methods and analysis

### Study design

ClusterVAP is an exploratory, observational, prospective, multicentre cross-sectional study designed to identify biologically and clinically meaningful subgroups (pneumoclusters) among patients with suspected VAP. The study will be conducted in ICUs across Sweden, France, Portugal, Denmark and the UK. The protocol follows the Standard Protocol Items: Recommendations for Interventional Trials 2013 recommendations for interventional and observational studies (online supplemental file 1).^8^

### Patient and public involvement statement

No patients were involved in the development of this research.

### Study setting

Participating centres include ICUs in Sweden (Skåne University Hospital, University Hospital of Umeå, Karolinska University Hospital), France (CHU Limoges, Pitié Salpêtrière Hospital), Portugal (Hospital de São Francisco Xavier), Denmark (Rigshospitalet) and the UK (King’s College Hospital). These centres were selected to ensure diversity in patient populations and clinical practice while maintaining capacity for BAL sampling and adherence to harmonised protocols.

### Eligibility criteria

Adult patients (>18 years) who are admitted to an ICU, are receiving mechanical ventilation and from whom BAL or mini-BAL is collected on clinical indication for bacterial culture will be screened for inclusion and recruited to the study.

### Inclusion criteria

- New or worsening clinical signs compatible with lower respiratory tract infection within the preceding 24 hours, defined as the treating clinician’s suspicion plus at least one of:
  - Body temperature >38°C or <36°C.
  - Visually purulent tracheal secretions.
  - Worsened oxygenation, demonstrated by the need to increase respiratory support to maintain the same partial pressure of oxygen/peripheral capillary oxygen saturation (SpO₂) target, defined as any of the following:
    - Increase in fractional inspired oxygen (FiO₂) by ≥0.1 if baseline FiO₂ ≤0.5, or increase by ≥0.15 if baseline FiO₂ >0.5.
    - Increase in positive end-expiratory pressure by ≥4 cmH₂O.
  - Request for chest radiograph to investigate infection or new infiltrate.
  - Initiation of antibiotic therapy targeting lower respiratory tract infection.
- Age 18 years or older at enrolment.

### Exclusion criteria

- BAL or mini-BAL collected solely for screening without suspicion of infection.
- Concurrent enrolment in the VAPmarkers study, which is reserved for external validation.

### Consent

Because patients eligible for inclusion are critically ill and typically lack decision-making capacity at the time of sampling, and the risk of harm or discomfort anticipated by participation in the study infers minimal risk, consent will be waived or deferred where permitted by local legislation and approved by relevant review boards. If deferred, written informed consent will be sought from the patient; if this is not possible, consent will be obtained from a legally authorised representative in accordance with site specific requirements. All consent procedures will be documented, and any additional provisions for the use of biological specimens and associated data will comply with national and institutional policies.

### Sampling and laboratory procedures

BAL or mini-BAL will be performed according to local standard of care using either bronchoscopy or an unguided catheter. The sample will be split for routine microbiology and for study-specific proteomic analysis using liquid chromatography tandem mass spectrometry (LC MS/MS). After routine microbiological analysis, the remaining supernatant will be centrifuged at 2000 g for 5 min, aliquoted into sterile nuclease-free vials (target 2 mL, minimum 1 mL) and stored at −20°C to −80°C. The time from sample collection to freezing should be kept to a minimum and must not exceed 12 hours. Samples will be shipped on dry ice in batches to Lund University for proteomic analysis.

Proteomic profiling will be performed using LC MS/MS with data independent acquisition. Quality control will include pooled reference samples and replicate injections. Total protein concentration will be measured by a colourimetric assay, and selected proteins will be quantified by ELISA.

Biological samples will be stored under controlled conditions for up to 10 years to allow confirmatory analyses, after which they will be destroyed.

### Outcomes

#### Primary outcome

- Identification of pneumoclusters among patients with suspected VAP using unsupervised consensus clustering of BAL proteomic data alone and in combination with clinical and microbiological variables.

#### Secondary outcomes

- Comparison of 30-day clinical outcomes across clusters, including: All-cause mortality, ventilator-free days, antibiotic-free days, ICU-free days and hospital-free days.
- Derivation of candidate protein biomarkers for pragmatic cluster assignment.

### Sample size

As an exploratory clustering study, a formal power calculation is not applicable. Based on anticipated heterogeneity and the expectation of four to six major clusters, the target sample size is 400 patients. This number balances scientific aims with the resource-intensive nature of proteomic analysis and is considered sufficient to identify clinically meaningful clusters while allowing for internal validation.

### Data collection and management

Data will be recorded in electronic case report forms hosted in REDCap at Lund University, with access restricted to authorised study personnel. The first form will capture enrolment and sampling details; the second, completed at least 30 days after enrolment, will include demographics, baseline clinical data, microbiology results and outcomes. Built-in range checks and logic controls will support data quality. All data will be pseudonymised using site-specific sequential codes, with re-identification keys stored securely at each site.

### Statistical analysis

Proteome-driven unsupervised consensus clustering will be used to discover pneumoclusters that are then described using clinical, microbiological and radiological features. Proteomic data processing will include analysing raw DIA files (dia-PASEF on Bruker timsTOF) with DIA-NN (V.1.8.1) against the UniProt human reference proteome. False discovery rate (FDR) will be controlled at 1% at both precursor and protein levels using a target–decoy strategy. Proteins with >70% missing values across all samples will be removed. Retention-time–dependent normalisation followed by median normalisation will be applied.

Unsupervised consensus clustering with resampling will be applied to define stable clusters, with cluster number determined by cumulative distribution function and silhouette width. Differential abundance will be assessed using limma. Multiple testing will be controlled with Benjamini-Hochberg correction and proteins will be considered significant at adjusted p<0.05 and |log₂ fold-change|>1.5. Gene set enrichment will be performed using clusterProfiler against Gene Ontology and Reactome collections with FDR<0.01.

30-day outcomes, including mortality and days free of ventilation, intensive care, hospitalisation and antibiotics, will be compared across clusters to characterise distinct clinical trajectories. Missing proteomic data will lead to exclusion from clustering; missing clinical variables will be handled by complete case analysis for relevant comparisons.

Candidate protein biomarkers that enable pragmatic assignment to clusters will be derived using differential expression methods and feature selection methods, with the intention of informing future validation with receiver operating characteristic (ROC) curves in separate patient cohorts and, ultimately, clinical application. Biomarker validation is planned in an independent external prospective observational study (VAPmarkers; ClinicalTrials.gov: NCT05117125). Validation will rely on targeted quantification of selected proteins using ELISA or equivalent immunoassays, and diagnostic and prognostic performance will be assessed using ROC curves.

To evaluate the robustness of our findings, we will perform several prespecified sensitivity analyses. First, we will repeat the clustering and differential abundance analyses using a stricter protein-completeness threshold (requiring ≥90% quantification across samples) to determine whether pneumocluster structure is sensitive to missingness. We will also reassess key analyses restricted to microbiology-confirmed cases with pneumonia in order to evaluate the effect of potential misclassification. In addition, we will repeat the unsupervised clustering using data from the largest enrolling site alone to examine whether cluster formation persists independently of between-centre heterogeneity. Finally, to assess potential influence of preanalytical handling, we will analyse the relationship between time from collection to freezing and proteomic structure, and repeat the main analyses after excluding samples with the longest delays to ensure that freezing time does not bias cluster allocation or biomarker identification.

## Ethics and dissemination

Ethical approval will be obtained from the relevant research ethics committees or institutional review boards at all participating sites before study initiation. The Swedish Ethical Review Authority has approved the study protocol (Dnr 2025-04564-01) including waived consent. The study will be conducted in accordance with the principles of the Declaration of Helsinki and applicable national regulations governing research involving human participants.

The results of the study will be disseminated through publication in peer-reviewed journals and presentation at national and international scientific meetings. Statistical code and study protocol will be made available alongside the main report to support transparency and reproducibility. Raw proteomic data from ClusterVAP will be deposited in the PRIDE repository (ProteomeXchange) after publication. De-identified datasets may be shared on reasonable request, subject to approval by the relevant ethics committees and data transfer agreements.

### Strengths and limitations of this study

- Multicentre design across five European countries enhances generalisability of findings.
- Use of bronchoalveolar lavage proteomics provides direct insight into local host response at the site of disease.
- Integration of molecular, clinical and microbiological data enables comprehensive characterisation of patient subgroups.
- Observational cross-sectional design precludes causal inference and limits assessment of temporal dynamics.
- Resource-intensive proteomic analysis may constrain scalability and external validation.

## Supplementary material

10.1136/bmjresp-2025-003830 online supplemental file 1

## Data Availability

Data sharing not applicable as no datasets generated and/or analysed for this study.

## References

[1] ECDC (2019). European centre for disease prevention and control. healthcare-associated infections acquired in intensive care units.

[2] Chastre J, Fagon JY (2002). Ventilator-associated Pneumonia. Am J Respir Crit Care Med.

[3] Klompas M (2007). Does This Patient Have Ventilator-Associated Pneumonia?. JAMA.

[4] Kalil AC, Metersky ML, Klompas M (2016). Management of Adults With Hospital-acquired and Ventilator-associated Pneumonia: 2016 Clinical Practice Guidelines by the Infectious Diseases Society of America and the American Thoracic Society. Clin Infect Dis.

[5] Fernando SM, Tran A, Cheng W (2020). Diagnosis of ventilator-associated pneumonia in critically ill adult patients—a systematic review and meta-analysis. Intensive Care Med.

[6] Martin-Loeches I, Bos LD, Povoa P (2015). Tumor necrosis factor receptor 1 (TNFRI) for ventilator-associated pneumonia diagnosis by cytokine multiplex analysis. Intensive Care Med Exp.

[7] Gromelsky Ljungcrantz E, Askman S, Sjövall F (2025). Biomarkers in lower respiratory tract samples in the diagnosis of ventilator-associated pneumonia: a systematic review. Eur Respir Rev.

[8] Chan A-W, Tetzlaff JM, Altman DG (2013). SPIRIT 2013 statement: defining standard protocol items for clinical trials. Ann Intern Med.

