# Supplementary material for: ClusterVAP: study protocol for multicentre proteomic endotyping of ventilator-associated pneumonia
Source: BMJ Open Respir Res. 2026 Mar 9;13(1):e003830. doi: 10.1136/bmjresp-2025-003830 (PMC12983971; doi:10.1136/bmjresp-2025-003830)
Supplement: online supplemental file 1 [file bmjresp-13-1-s001.docx]

# Supplementary file 1: SPIRIT 2013 Checklist

# ClusterVAP: Study protocol for multicentre proteomic endotyping of ventilator-associated pneumonia

# Authors and Affiliations

Authors:
Fredrik Sjövall, Pedro Póvoa, Johan Petersson, Despoina Koulenti, Ana Catalina Hernandez Padilla, Julien Vaidie, Alicia Lind, Frederik Boëtius Hertz, Magnus Paulsson*,

*Corresponding author: magnus.paulsson@med.lu.se

| SPIRIT Item | Description | Location in Manuscript |
| --- | --- | --- |
| 1. Title | See SPIRIT guidance for full item description. | Title, page 1 |
| 2a. Trial registration | See SPIRIT guidance for full item description. | NCT07245888, page 3 |
| 2b. WHO Trial Registration Data Set | See SPIRIT guidance for full item description. | NA |
| 3. Protocol version | See SPIRIT guidance for full item description. | Patient enrolment has not not started. |
| 4. Funding | See SPIRIT guidance for full item description. | Funding statement, page 15 |
| 5a. Roles and responsibilities | See SPIRIT guidance for full item description. | Authors’ contributions, page 14 |
| 5b. Sponsor contact information | See SPIRIT guidance for full item description. | Corresponding author |
| 5c. Role of sponsor and funders | See SPIRIT guidance for full item description. | Funding statement, page 15 |
| 5d. Committees | See SPIRIT guidance for full item description. | NA |
| 6a. Background and rationale | See SPIRIT guidance for full item description. | Introduction section, page 5-7 |
| 6b. Explanation for choice of comparators | See SPIRIT guidance for full item description. | This is an observational, exploratory study without an interventional component; therefore, no active comparator group is included. All enrolled patients will receive standard care as determined by the treating clinical team. The study aims to characterise biological and clinical heterogeneity among patients with suspected ventilator-associated pneumonia rather than to compare treatment strategies. |
| 7. Objectives | See SPIRIT guidance for full item description. | Outcomes, page 11 |
| 8. Trial design | See SPIRIT guidance for full item description. | Study design, page 8 |
| 9. Study setting | See SPIRIT guidance for full item description. | Study setting, page 8 |
| 10. Eligibility criteria | See SPIRIT guidance for full item description. | Eligibility criteria, page 8 |
| 11a. Interventions | See SPIRIT guidance for full item description. | This is an observational study; no experimental intervention will be administered. All patients will receive standard care as determined by the treating clinical team. Bronchoalveolar lavage (BAL) or mini-BAL sampling will be performed only when clinically indicated for suspected lower respiratory tract infection, following local standard procedures. |
| 11b. Discontinuing/modifying interventions | See SPIRIT guidance for full item description. | NA |
| 11c. Adherence strategies | See SPIRIT guidance for full item description. | NA |
| 11d. Concomitant care | See SPIRIT guidance for full item description. | NA |
| 12. Outcomes | See SPIRIT guidance for full item description. | Outcomes, page 11 |
| 13. Participant timeline | See SPIRIT guidance for full item description. | This is a cross-sectional study with no longitudinal intervention or follow-up beyond 30 days. |
| 14. Sample size | See SPIRIT guidance for full item description. | Sample size, page 11 |
| 15. Recruitment | See SPIRIT guidance for full item description. | Recruitment, page 8-9 |
| 16a. Sequence generation | See SPIRIT guidance for full item description. | NA |
| 16b. Allocation concealment | See SPIRIT guidance for full item description. | NA |
| 16c. Implementation | See SPIRIT guidance for full item description. | NA |
| 17a. Blinding | See SPIRIT guidance for full item description. | NA |
| 17b. Unblinding | See SPIRIT guidance for full item description. | NA |
| 18a. Data collection methods | See SPIRIT guidance for full item description. | Data collection, page 12 |
| 18b. Retention strategies | See SPIRIT guidance for full item description. | NA |
| 19. Data management | See SPIRIT guidance for full item description. | Data management, page 12 |
| 20a. Statistical methods | See SPIRIT guidance for full item description. | Statistical analysis, page 12 |
| 20b. Additional analyses | See SPIRIT guidance for full item description. | NA |
| 20c. Missing data | See SPIRIT guidance for full item description. | Page 12-13 |
| 21a. Data monitoring committee | See SPIRIT guidance for full item description. | NA |
| 21b. Interim analyses | See SPIRIT guidance for full item description. | NA |
| 22. Harms | See SPIRIT guidance for full item description. | NA |
| 23. Auditing | See SPIRIT guidance for full item description. | NA |
| 24. Ethics approval | See SPIRIT guidance for full item description. | Page 13 |
| 25. Protocol amendments | See SPIRIT guidance for full item description. | NA |
| 26a. Consent | See SPIRIT guidance for full item description. | Page 13 |
| 26b. Additional consent provisions | See SPIRIT guidance for full item description. | NA |
| 27. Confidentiality | See SPIRIT guidance for full item description. | NA |
| 28. Declaration of interests | See SPIRIT guidance for full item description. | Competing interests, page 15 |
| 29. Data access | See SPIRIT guidance for full item description. | Page 13 |
| 30. Ancillary and post-trial care | See SPIRIT guidance for full item description. | NA |
| 31a. Dissemination policy | See SPIRIT guidance for full item description. | Dissemination, page 13 |
| 31b. Authorship eligibility | See SPIRIT guidance for full item description. | Authors’ contributions, page 15 |
| 31c. Public access to protocol and data | See SPIRIT guidance for full item description. | Page 13 |
| 32. Informed consent materials | See SPIRIT guidance for full item description. | NA |
| 33. Biological specimens | See SPIRIT guidance for full item description. | Page 11 |
